# TACE performed in patients with a single nodule of Hepatocellular Carcinoma

**DOI:** 10.1186/1471-2407-14-601

**Published:** 2014-08-19

**Authors:** Eleonora Terzi, Fabio Piscaglia, Ludovica Forlani, Cristina Mosconi, Matteo Renzulli, Luigi Bolondi, Rita Golfieri, BLOG-Bologna Liver Oncology Group, S.Orsola-Malpighi Hospital, University of Bologna, Bologna, Italy

**Affiliations:** Division of Internal Medicine, Department of Digestive Disease and Internal Medicine, Sant’Orsola-Malpighi Hospital, University of Bologna, Via Albertoni 15, 40138 Bologna, Italy; Radiology Unit, Department of Digestive Disease and Internal Medicine, Sant’Orsola-Malpighi General and University Hospital, Bologna, Italy

**Keywords:** Hepatocellular carcinoma, Transarterial chemoembolization, Tumor radiological response

## Abstract

**Background:**

Patients with single hepatocellular carcinoma (HCC) usually undergo transarterial chemoembolization (TACE) if they are not candidates for curative surgical or ablative therapy. The primary aim of the study was to assess the overall survival and clinical determinants of survival in patients with single HCC who underwent TACE. The secondary aims were tumor response, local and distant recurrence rates, time to recurrence and the impact of TACE on liver function.

**Methods:**

The outcomes of 148 consecutive patients with single HCC who underwent TACE from January 2004 to December 2009 were retrospectively analyzed.

**Results:**

Complete response (CR) was observed in 95/148 (64%) patients and a partial response (PR) in 39 (26%) patients. The recurrence rate was 27%, 42% and 65% at 6, 12 and 24 months, respectively. The day after TACE, 56 (38%) patients had a Child-Pugh increase ≥1 and 93 (63%) had a MELD increase ≥1. Median survival was 36.0 months with 1-, 3- and 5-year survival rates of 85%, 50% and 26%, respectively. Bland portal thrombosis was not seen to have any impact at univariate survival analysis; however, a slight impairment of PS (PS-1) in small tumors had some, although minor, impact on prognosis. Factors associated with shorter survival at multivariate analysis were tumor >5 cm, absence of CR, ascites, alpha-fetoprotein (AFP) ≥14.5 ng/mL and a MELD increase ≥1.

**Conclusions:**

Transarterial chemoembolization is a valid treatment option in patients with single HCC not suitable for curative treatment. Bland PVT has no major impact on survival and a slight impairment of PS attributable to cirrhosis in patients within the Milan criteria should not preclude the use of TACE.

## Background

Curative treatment is considered the first choice treatment for patients with single hepatocellular carcinoma (HCC) according to the international guidelines [1]. In particular, liver transplant (LT) is recommended in patients within the Milan criteria (MC) [2], and surgical resection or ablation in patients not suitable for LT [3]. In clinical practice, however, patients with a single tumor unsuitable for curative treatment are usually treated with transarterial chemoembolization (TACE) on the basis of a clinical judgment. In fact, according to the “stage migration” concept, patients who cannot receive the recommended treatment allocation within their stage should be offered treatment with the next most suitable option within the same stage or the next stage [1]. Transarterial chemoembolization is a well-established treatment for HCC and the current guidelines recommend TACE as a first line non-curative treatment for intermediate stage patients with multinodular asymptomatic tumors without vascular invasion or extrahepatic spread [1]. Nonetheless, the percentage of patients with single HCC who routinely underwent TACE is higher than 40% in many studies [4–6].

The primary endpoint of the present study was to evaluate the overall survival and clinical determinants of survival, including the presence of bland portal vein thrombosis (PVT) and slight impairment of performance status (PS), in patients with a single nodule of HCC who underwent TACE and could not undergo curative treatment.

The secondary end points were tumor response at 1 month, local and distant recurrence rates, time to recurrence and impact of TACE on liver function.

### Patients and methods

#### Patient population

The present retrospective analysis was based on a database of 902 consecutive patients who underwent TACE as a first line treatment between January 2004 and December 2009 in the Interventional Radiology Unit of Sant’Orsola-Malpighi Hospital in Bologna after a multidisciplinary team (MDT) discussion. The analysis of the follow-up was closed in May 2012 in order to have at least 30 months of follow-up for each patient. The inclusion criteria for enrollment in the study was: (1) diagnosis of single HCC according to the European Association for the Study of the Liver/American Assoication for the Study of Liver Diseases (EASL/AASLD) criteria [7, 8]; (2) Child-Pugh-Turcotte (CPT) hepatic function A or B; (3) PS 0 or 1 and (4) first conventional TACE performed between January 2004 and December 2009. The exclusion criteria were: (1) the absence of at least one imaging control (CT: Computed Tomography and/or MRI: Magnetic Resonance Imaging) before and after TACE treatment; (2) multiple HCC nodules; (3) portal branch/hepatic vein tumor invasion or extrahepatic spread (4) Child-Pugh hepatic function C; (5) PS ≥2; (6) previous treatment for HCC and (7) non-conventional TACE treatment (DC-Beads, mixed treatments or radioembolization).

Portal vein thrombosis was considered to be bland or neoplastic based on definite criteria previously reported by our group [9].

**Figure 1:**
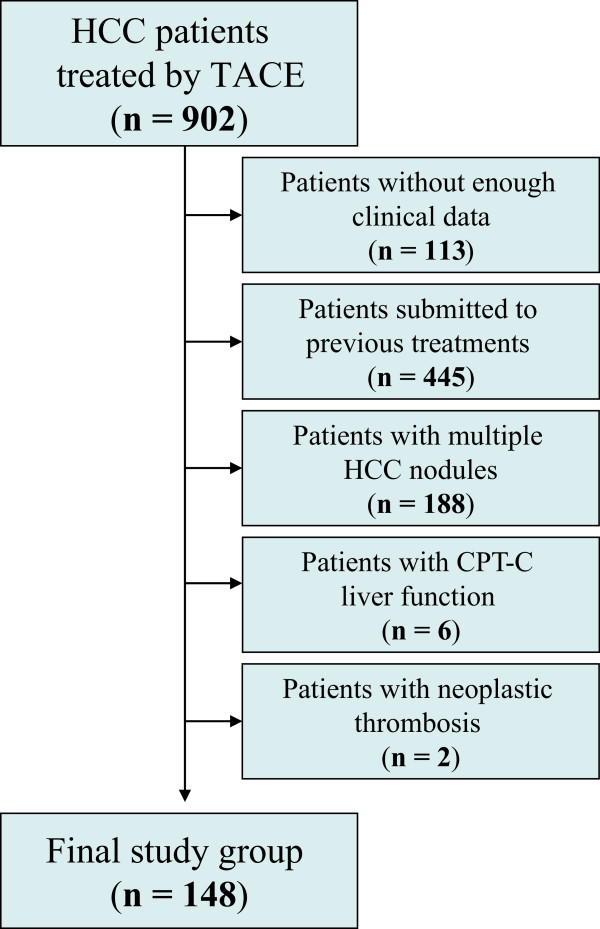
**Flow chart of the study population.**

In the series of consecutive patients, one hundred and forty-eight patients fulfilled the inclusion criteria, and were therefore selected as the cohort for the study (Figure 1).

The study protocol complied with the provision of the Good Clinical Practice guidelines and the Declaration of Helsinki and was approved by the Institutional Review Board S.Orsola-Malpighi hospital. Collection of informed consents was waived given the retrospective nature of the study.

## Methods

### TACE protocol and technical procedure

In our clinical practice, HCC treatment for patients with single HCC follows the BCLC staging system [10] but each case is discussed in MDT meetings and individually tailored, according to the considerations recently included in the recommendations of the Italian Association for the Study of the Liver [11].

Transarterial chemoembolization treatment was performed in single nodules if curative treatment was not feasible due to tumor size, tumor location, technical applicability of treatment, severity of liver dysfunction, presence of portal hypertension, presence of comorbidities and their severity, and individual consent for specific treatment.

Before treatment, baseline clinical evaluation, laboratory tests, chest X-ray and tumor stage were assessed in all patients. Very few patients underwent TACE despite a CPT function of B8-B9, which usually contraindicates TACE due to the risk of irreversible terminal liver failure. Those patients were treated because they were on the waiting list for liver transplantation and they could undergo salvage liver transplantation in case of liver failure. At admission, daily living abilities were assessed and PS was calculated [12]. According to the guidelines [1], all patients with compromised abilities (PS 1) were classified as being into advanced tumor stage (BCLC-C) irrespective of their origin (given the extreme difficulty and subjectivity to ascribe such complaints either to the underlying cirrhosis or to the occurrence of cancer).

Conventional TACE was carried out by selective catheterization of the hepatic arteries feeding the lesions; in the majority of patients, superselective or selective TACE was carried out using a highly flexible coaxial microcatheter (2.7-2.8 Fr Progreat™ Terumo or Renegade™ Hi-flo Boston Scientific) passed through a 4Fr catheter previously placed in the hepatic artery. For selective transarterial chemoembolization, the tip of the microcatheter was placed into the hepatic arterial branch afferent to the segment where the tumor was located. In superselective TACE, the tip of the catheter was additionally advanced into the sub-segmental branches feeding the nodule [13]. A lobar technique was carried out in the case of a nodule fed by multiple arteries or when the selective/superselective catheterization of the feeding artery was not technically feasible. All patients with PVT underwent a selective/superselective procedure.

After microcatheter placement, a mixture of epirubicin (Farmorubicin; Pfizer, Latina, Italy) and iodized oil (Lipiodol; Guerbet, Milan, Italy) was injected under fluoroscopic control, followed by embolization using Spongel (Gelitaspongel®) particles until complete blockage of the tumor-feeding vessels was demonstrated. When the interventional radiologist was aware of being unable to achieve complete tumor embolization in only a single TACE session (for example, due to the use of the maximum dose of Epirubicin allowed), the treatment was split into two sessions approximately 1 month apart. In the present study, the two treatments were considered as only one treatment cycle. The mean chemotherapeutic agent dose administered per treatment was approximately 40 mg of epirubicin (range, 20–75 mg) and the mean Lipiodol dose administered was approximately 8 mL (range, 4–15 mL). Upon demonstration of a persistent viable tumor or intrahepatic distal recurrence at imaging follow-up, TACE was repeated “on demand”.

### Assessment of tumor radiological response and follow-up

Patients underwent imaging assessment (quadriphasic CT or dynamic MRI) one month after TACE in order to evaluate the radiological response according to clinical practice. For the purpose of the study, all patients were evaluated according to the modified Response Evaluation Criteria in Solid Tumors (mRECIST) [14]. The response was considered complete (CR) when a dense homogeneous Lipiodol uptake with complete disappearance of any intratumoral enhancement was observed in the target lesion at CT scan or when no enhancement of the target nodule was observed at Dynamic MRI [14]. The other radiological responses were considered to be partial response (PR), progressive disease (PD) and stable disease (SD) according to the mRECIST criteria [14].

In all patients with a CR, a follow-up CT or MRI at 3–6 months was performed. A plain chest X-ray or chest CT were additionally utilized in the follow-up. For the assessment of overall survival, patient follow-up was carried out at the closure time of the study, at the time of death or at the last inpatient/outpatient clinical evaluation when no additional information was available (patients lost to follow-up). For the assessment of recurrence-free survival, patients were checked at the time of recurrence or death, at liver transplant (if performed) or at the last inpatient/outpatient clinical evaluation when no additional information was available (patients lost to follow-up).

### Statistical analysis

Continuous variables were reported as medians and ranges. Comparisons among groups were calculated using non-parametric tests (Mann–Whitney and Wilcoxon). Categorical variables were compared using the *χ*^2^ test. All tests were considered significant at P <0.05. Overall survival was defined as the time interval between TACE and death or the date of the last follow-up. Univariate analysis was carried out in order to identify the factors predicting survival. Survival curves were computed according to Kaplan-Meier methods and were compared using log rank tests. Variables with P <0.1 in the univariate analysis were entered into a stepwise Cox regression model (conditional backward selection) to assess their impact as independent predictive factors. For patients who dropped out of the study, survival could be calculated by requesting the living status or time of death from the registry offices of the patients’ hometowns, making them assessable for the survival analysis.

Analysis of the data was carried out using SPSS statistical analysis software (SPSS Inc., Chicago, Illinois, USA, 1999).

## Results

Transarterial chemoembolization was the primary treatment after diagnosis of HCC in 148 patients with a single nodule of HCC who were not eligible for curative treatment (final study group) (Figure 1); their characteristics are reported in Tables 1 and 2. Transarterial chemoembolization was performed once in 80 patients (54%), twice in 44 patients (30%), three times in 17 patients (11%) and 4 times in 7 patients (5%). All patients with hepatitis B virus (HBV)-related cirrhosis received oral antiviral treatment as appropriate.

**Table 1:** **Baseline demographic, clinical and tumor characteristics of the whole patient cohort before TACE treatment**

Variable	Study population (n = 148)
**Gender male, n (%)**	104 (70)
**Age, median (years) (range)**	64 (36–84)
**Cause of disease, n (%)**	
HBV	14 (9)
HCV	85 (57)
Alcohol	19 (13)
Multiple etiologies	17 (12)
Unknown	12 (8)
Other	1 (1)
**Lesion location, n (%)**	
Right lobe	110 (74)
Left lobe	38 (26)
**TACE Selectivity, n (%)**	
Lobar	10 (7)
Selective	73 (49)
Superselective	65 (44)
**Tumor size, median (cm) (range)**	3.0 (0.8 – 15.0)
**Milan criteria within, n (%)** ^*^	135 (91)
**Portal vein thrombosis, n (%)**	
Absent	131 (88)
Segmental bland thrombosis	4 (3)
Lobar bland thrombosis	13 (9)
**Serum AFP, median (ng/mL) (range)**	14.5 (1.0 – 39576.0)
**Ascites, n (%)**	
Absent	120 (81)
Slight – Moderate	23 (16)
Severe – Refractory	5 (3)
**Encephalopathy, n (%)**	
Absent	145 (98)
Slight	3 (2)
**Serum total bilirubin, median (mg/dL) (range)**	1.34 (0.30 – 10.67)
**Serum albumin, median (g/dL) (range)**	3.60 (2.10 – 5.00)
**Serum INR, median (range)**	1.28 (1.00 – 1.97)
**Hepatic function, n (%)**	
CPT-A	92 (62)
CPT-B	56 (38)
**Performance status, n (%)**	
0	133 (90)
1	15 (10)
**BCLC stage, n (%)**	
0	16 (11)
A	104 (70)
B	13 (9)
C	15 (10)
**MELD score, median (range)**	11 (6 – 24)

^*^patients with HCC ≤5 cm.

*Abbreviations*: *HBV* hepatits B virus, *HCV* hepatitis C virus, *TACE* transarterial chemoembolization, *AFP* Alpha-fetoprotein, *INR* international normalized ratio, *CPT* Child-Pugh-Turcotte score, *BCLC* Barcelona Clinic Liver Cancer, *MELD* Model for end stage liver disease.

**Table 2:** **Clinical and tumor characteristics of the whole patient cohort before TACE treatment according to BCLC tumor stage**

Variable	BCLC-0 (n = 16)	BCLC-A (n = 104)	BCLC-B (n = 13)	BCLC-C (n = 15)
**Within Milan Criteria, n (%)** ^*^	16 (100)	104 (100)	0	15 (100)
**Tumor size, median (cm) (range)**	1.3 (1.0 – 1.9)	3.0 (0.8 – 5.0)	6.0 (5.1 – 15.0)	3.0 (1.0 – 4.8)
**Portal vein thrombosis, n (%)**				
Absent	14 (88)	94 (90)	11 (85)	12 (80)
Segmental bland thrombosis	0	3 (3)	0	1 (7)
Lobar bland thrombosis	2 (12)	7 (7)	2 (15)	2 (13)
**Hepatic function pre TACE, n (%)**				
CPT-A	16 (100)	58 (56)	8 (62)	10 (67)
CPT-B	0	46 (44)	5 (38)	5 (33)
**Performance status pre TACE, n (%)**				
0	16 (100)	104 (100)	13 (100)	0
1	0	0	0	15 (100)

Percentages should be read as columns.

^*^patients with HCC ≤5 cm.

*Abbreviations*: *BCLC* Barcelona Clinic Liver Cancer, *TACE* transarterial chemoembolization, *CPT* Child-Pugh-Turcotte score.

### Tumor response at 1 month

A CR at one month was obtained in 95/148 (64%) patients, a PR in 39 (26%), SD in 1 patient and PD in 10 (7%). Three patients were not evaluable (1 underwent radiofrequency as a complementary treatment after TACE and 2 received a liver transplant within 1 month after the procedure, before the CT).

At univariate analysis of pre-TACE clinical and tumoral variables to predict a complete radiological response (CR vs. non-CR), only tumor size was found to be a statistically significant predictor of complete response (Table 3), in particular, a tumor diameter ≤3 cm (*P* = 0.017) and, more significantly, ≤5 cm (tumors within the Milan criteria, *P* = 0.004). A trend towards higher pre-TACE values of alpha-fetoprotein (AFP) was found in incomplete responders.

**Table 3:** **Clinical and tumor characteristics of the whole patient cohort before TACE treatment according to tumor response**

Variable	CR (n = 95)	Non-CR (n = 50)	*P*
**Gender male, n (%)**			0.122
Male	71 (70)	31 (30)	
Female	24 (56)	19 (44)	
**Age, median (years) (range)**	63 (36–83)	67.5 (45–84)	0.099
**Cause of disease, n (%)**			0.928
HBV	7 (50)	7 (50)	
HCV	54 (66)	28 (34)	
Alcohol	15 (79)	4 (21)	
Multiple etiologies	9 (53)	8 (47)	
Unknown	9 (75)	8 (25)	
Other	1 (100)	3 (0)	
**Lesion location, n (%)**			0.453
Right lobe	72 (67)	35 (33)	
Left lobe	23 (61)	15 (39)	
**TACE selectivity, n (%)**			0.992
Lobar	5 (50)	5 (50)	
Selective	45 (63)	26 (37)	
Superselective	45 (70)	19 (30)	
**Tumor size, n (cm) (%)**			**0.017**
≤3.0	65 (73)	24 (27)	
>3.0	30 (54)	26 (46)	
**Milan criteria, n (%)**			**0.004**
Within (≤5.0 cm)	92 (69)	41 (31)	
Beyond (>5.0 cm)	3 (25)	9 (75)	
**Portal vein thrombosis, n (%)**			0.789
Absent	83 (65)	45 (35)	
Bland thrombosis	12 (71)	5 (29)	
**Serum AFP, median (ng/mL) (range)**	13.0 (1.0 – 10000.0)	21.0 (2.0 – 39576.0)	0.094
**Ascites, n (%)**			1.000
Absent	77 (66)	40 (34)	
Present	18 (64)	10 (36)	
**Encephalopathy, n (%)**			1.000
Absent	93 (66)	49 (34)	
Slight	2 (67)	1 (33)	
**Serum total bilirubin, median (mg/dL) (range)**	1.39 (0.30 – 7.56)	1.24 (0.35 – 7.72)	0.594
**Serum albumin, median (g/dL) (range)**	3.60 (2.10 – 5.00)	3.75 (2.40 – 4.90)	0.064
**Serum INR, median (range)**	1.29 (1.00 – 1.83)	1.23 (1.00 – 1.97)	0.365
**Hepatic function, n (%)**			0.155
CPT-A	55 (61)	35 (39)	
CPT-B	40 (73)	15 (27)	
**Performance status, n (%)**			0.775
0	86 (66)	44 (34)	
1	9 (60)	6 (40)	
**BCLC stage, n (%)**			0.077
0 – A	83 (70)	35 (30)	
B	3 (25)	9 (75)	
C	9 (60)	6 (40)	
**MELD score, median (range)**	12 (7 – 19)	11 (6 – 24)	0.637

Three patients not evaluable for tumor response were excluded from the analysis. Percentages should be read as rows.

*Abbreviations*: *CR* complete response, *HBV* hepatits B virus, *HCV* hepatitis C virus, *TACE* transarterial chemoembolization, *AFP* Alpha-fetoprotein, *INR* international normalized ratio, *CPT* Child-Pugh-Turcotte score, *BCLC* Barcelona Clinic Liver Cancer, *MELD* Model for end stage liver disease.

“P<0.05 are reported as bold numbers”.

### Local and distant recurrence after TACE

Out of 95 patients achieving a CR, 61 (64%) relapsed after a median time of 9 months (range 2–72), 28 (30%) did not relapse after a median follow-up of 13.5 months (range 2–53) and 6 patients were not evaluable. Out of the 61 patients who relapsed, 23 patients (38%) had local relapse after a median of 10 months (range 2–37), 23 (38%) had distant intrahepatic relapse after a median of 8 months (range 2–72) and 15 (24%) had both local and distant intrahepatic relapse after a median of 11 months (range 3–36). No patient developed extrahepatic spread before or concurrently with the detection of local or intrahepatic relapse. The overall recurrence rate in patients with complete response was 27%, 42% and 65% at 6, 12 and 24 months, respectively.

### Impact of TACE on laboratory tests the day after the procedure

A significant negative impact on liver function was observed the day after TACE treatment (Table 4). In particular, 56 (38%) patients suffered a CPT increase ≥1 point and 93 (63%) patients suffered a Model for end stage liver disease (MELD) score increase ≥1 point. Interestingly, a CPT and a MELD score increase ≥1 were not related to a lobar TACE procedure (*P* = 0.320 and *P* = 1.000, respectively).

**Table 4:** **Liver function parameters of the whole patient cohort before and one day after TACE procedure**

Variable	Pre-TACE	Post-TACE	*P*
**Ascites, n (%)**			0.564
Absent	120 (81)	116 (78)	
Present	28 (19)	32 (22)	
**Encephalopathy, n (%)**			1.000
Absent	145 (98)	145 (98)	
Slight	3 (2)	3 (2)	
**Serum total bilirubin, median (mg/dL) (range)**	1.34 (0.30 – 10.67)	1.85 (0.30 – 13.94)	**<0.001**
**Serum albumin, median (g/dL) (range)**	3.60 (2.10 – 5.00)	3.50 (2.10 – 4.50)	**<0.001**
**Serum creatinine, median (range)**	0.92 (0.50 – 1.73)	0.97 (0.57 – 2.27)	0.823
**Hepatic function, n (%)**			**0.010**
CPT-A	92 (62)	81 (55)	
CPT-B	56 (38)	61 (41)	
CPT-C	0	6 (4)	
**Hepatic function, n (%)**			0.054
CPT-A5	65 (44)	53 (36)	
CPT-A6	27 (18)	27 (18)	
CPT-B7	28 (19)	26 (18)	
CPT-B8	21 (14)	25 (17)	
CPT-B9	7 (5)	11 (7)	
CPT-C10	0	6 (4)	
**Serum INR, median (range)**	1.28 (1.00 – 1.97)	1.33 (1.00 – 2.00)	**0.003**
**MELD score, median (range)**	11 (6 – 24)	12 (7 –24)	**<0.001**

Percentages should be read as columns.

*Abbreviations*: *CPT* Child-Pugh-Turcotte score, *INR* international normalized ratio, *MELD* Model for end stage liver disease.

P<0.05 are reported as bold numbers.

**Figure 2:**
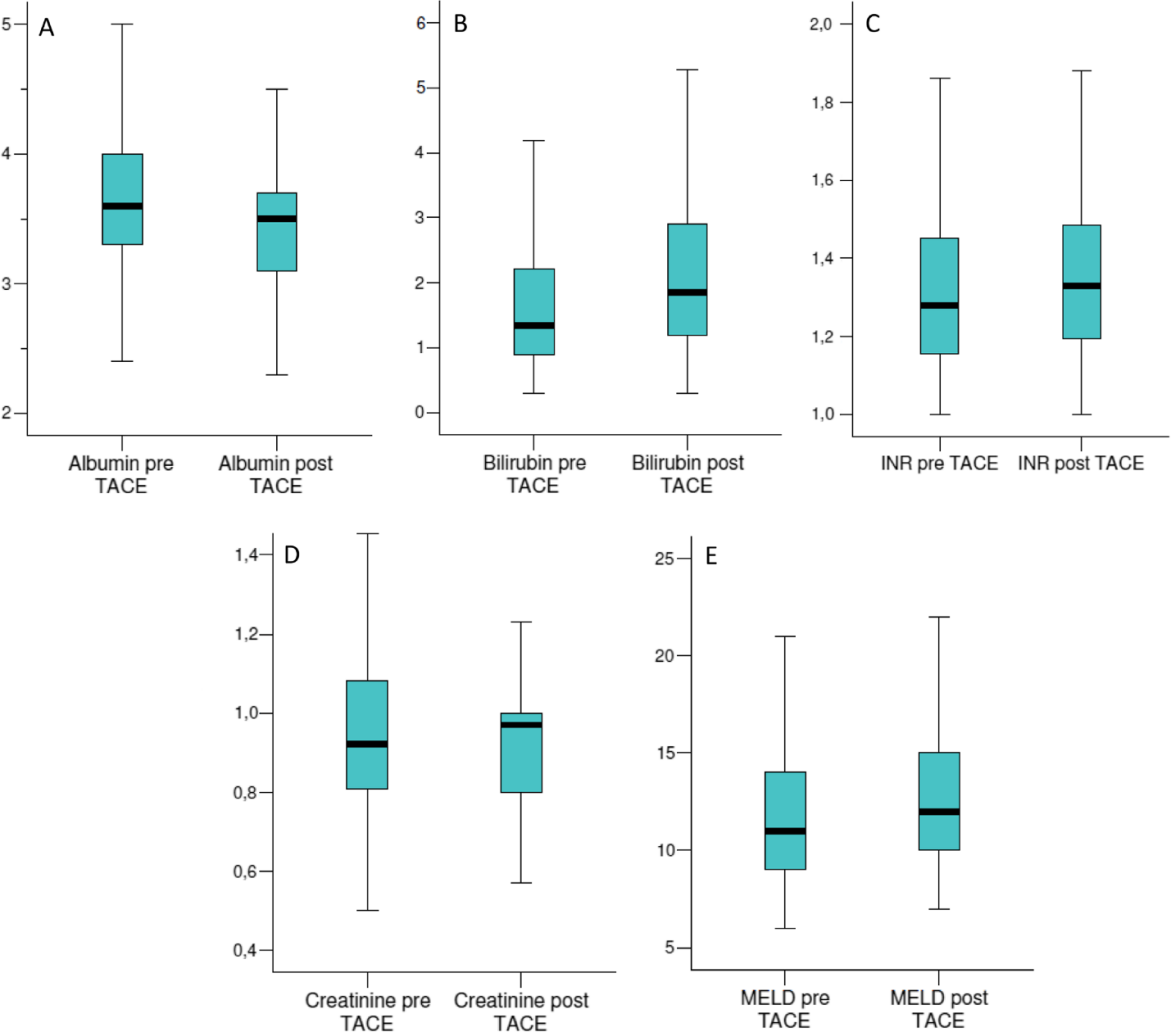
**Impact of TACE on laboratory tests one day after the procedure. (A)** Modification of serum albumin (*P* < 0.001); **(B)** modification of serum bilirubin (*P* < 0.001); **(C)** modification of serum INR (*P* = 0.003); **(D)** modification of serum creatinine (*P* = 0.823); **(E)** modification of MELD score (*P* < 0.001).

The impact of TACE on the serum levels of albumin, bilirubin, the international normalized ratio (INR), creatinine and the MELD score the day after TACE is reported in Figure 2. More in detail, the median serum albumin values decreased from 3.60 mg/dL (range 2.10-5.00) to 3.50 mg/dL (range 2.10–4.50; *P* < 0.001) whereas the median serum values of bilirubin, the INR and the MELD score increased from 1.34 mg/dL (range 0.30-10.67), 1.28 (range 1.00-1.97) and 11 (range 6–24) to 1.84 mg/dL (range 0.30-13.94; *P* <0.001), 1.33 (range 1.00-2.00; *P* = 0.003) and 12 (range 7–24; *P* < 0.001), respectively. The median serum value of creatinine increased from 0.92 mg/dL (range 0.50-1.73) to 0.97 mg/dL (0.57-2.27) but no statistical difference was observed (*P* = 0.823).

Patients with a CPT score increase ≥1 point after the first TACE underwent more often one single rather than multiple TACE courses (70% vs 30% of cases respectively, *P* = 0.006) in our routine clinical practice.

### Overall patient survival after TACE

Out of the 148 patients who underwent TACE, 79 (53%) died within the study period (January 2004 - May 2012) and 4 patients were lost to follow-up. The median overall follow-up of the entire study population was 44.0 months (95% CI = 33.5-54.5) with 1-, 3- and 5-year survival rates of 89%, 61% and 42%, respectively. If patients who underwent liver transplant (who generally were long-term survivors) were excluded (34 patients), the median overall follow-up decreased to 36.0 months (95% CI = 24.6–47.4) with 1-year, 3-year and 5-year survival rates of 85%, 50% and 26%, respectively. The median survival of patients within the Milan criteria was 37 months, compared to 6 months of those beyond the Milan criteria.

**Figure 3:**
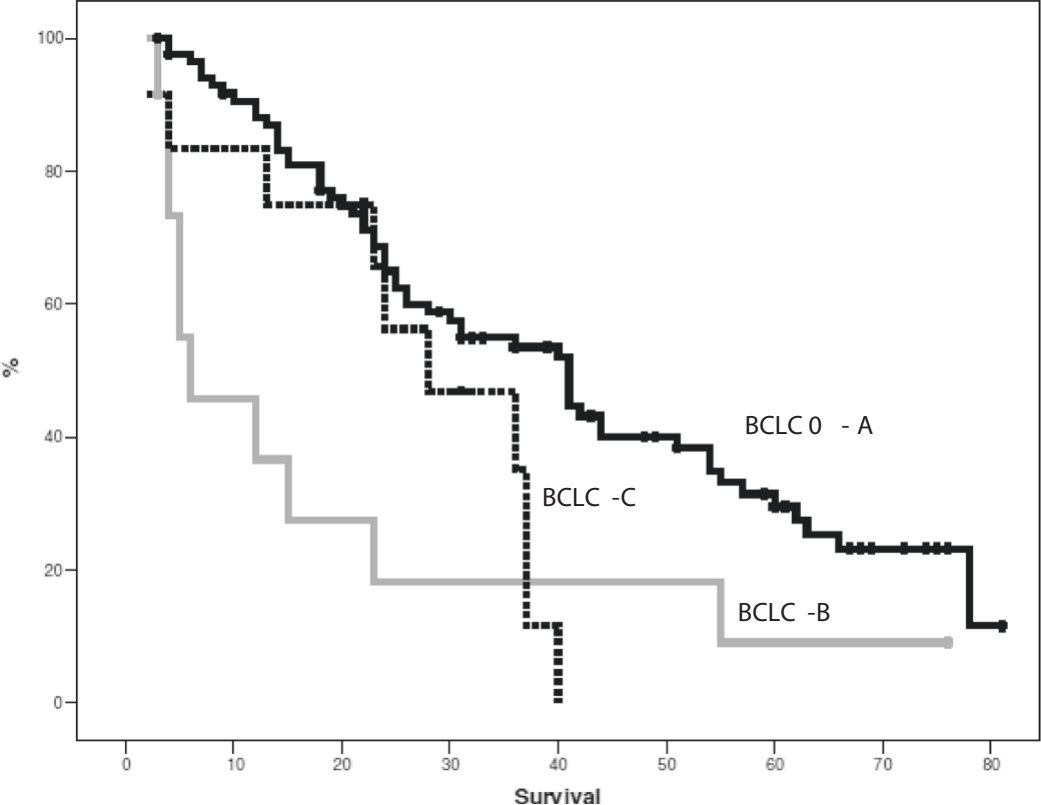
**Overall survival according to BCLC tumor stage.**

At univariate analysis, tumor characteristics (and particularly tumor size), some liver function parameters and the achievement of a complete radiological response (Table 5) had a statistical impact on survival. Interestingly, an increase of ≥1 point in the CPT or the MELD score the day after TACE was significantly associated with lower survival (*P* = 0.003) (Table 4). On the opposite the number of TACE was not associated with survival (*P* = 0.407). As expected, median survival was also influenced by the BCLC stage, but BCLC-B patients showed a lower median survival (6 months, *P* = 0.002) with respect to BCLC 0-A (41 months) and BCLC-C (28 months) patients (the latter categorized as BCLC-C only on the basis of PS-1, but with tumor burden within the MC) (Figure 3). Furthermore, the presence of bland segmental or lobar PVT had no impact on overall survival but a slight impairment in PS (PS-1) did have an impact on prognosis since survival in PS-1 patients (BCLC-C) was worse than in that of PS-0 patients within the MC (BCLC 0-A). Nonetheless, the impact of PS on survival was minor with respect to the tumor burden since survival in PS-1 patients (BCLC-C) was better than that in PS-0 patients beyond the MC (BCLC-B) (Tables 5 and 6, Figure 3).

**Table 5:** **Univariate survival analysis**

Variable	Survival (%)			Median survival (95% C.I.)	*P*
	1-yr	3-yr	5-yr		
**Gender**					
Male	84	45	29	26.0 (16.7 – 35.3)	0.975
Female	88	63	20	41.0 (34.9 – 47.1)	
**Age, yr**					
<64	80	35	26	24.0 (21.5 – 26.5)	0.453
≥64	88	59	26	40.0 (36.2 – 43.8)	
**Cause of disease**					
Alcohol	81	28	21	24.0 (22.5 – 25.5)	0.081
Non alcohol	86	56	27	40.0 (33.0 – 47.0)	
**TACE selectivity**					
Lobar	87	58	58	44.0 (0 –104.8)	0.823
Selective	87	51	29	36.0 (25.8 – 46.2)	
Superselective	82	49	27	26.0 (10.2 – 41.8)	
**Milan criteria**					
Within (≤5.0 cm)	90	54	28	37.0 (30.2 – 43.8)	**0.003**
Beyond (>5.0 cm)	46	18	10	6.0 (0 – 17.3)	
**Portal vein thrombosis**					
Absent	86	50	24	36.0 (24.0 – 48.0)	0.876
Bland thrombosis	78	52	39	36.0 (4.6 – 67.4)	
**AFP, ng/mL**					
<14.5	89	58	39	41.0 (23.0 – 59.0)	0.052
≥14.5	79	43	22	25.0 (19.5 – 30.5)	
**Ascites**					
Absent	92	55	28	37.0 (29.0 – 45.0)	**0.013**
Present	57	32	16	15.0 (3.0 – 27.0)	
**Serum total bilirubin, mg/dL**					
<1.34	92	62	31	41.0 (36.6 – 45.4)	**0.026**
≥1.34	74	32	18	23.0 (18.1 – 27.9)	
**Serum albumin, g/dL**					
<3.60	81	40	20	24.0 (18.3 – 29.6)	0.340
≥3.60	87	55	29	40.0 (32.4 – 47.6)	
**Serum INR**					
<1.28	89	58	27	40.0 (34.5 – 45.5)	0.211
≥1.28	80	40	23	25.0 (18.8 – 31.2)	
**Hepatic function**					
CPT-A	93	60	26	40.0 (36.1 – 43.9)	0.075
CPT-B	83	47	22	21.0 (15.3 – 26.7)	
**Hepatic function. Pts Milan in patients**					
CPT-A	97	60	31	41.0 (37.2 – 44.8)	0.053
CPT-B	74	35	22	21.0 (14.3 – 27.7)	
**Performance status**					
0	92	61	34	36.0 (24.7 – 47.3)	0.080
1	78	41	18	28.0 (11.2 – 44.7)	
**PS. Patients Milan In**					
0	91	55	31	41.0 (31.5 – 50.5)	**0.029**
1	83	50	0	28.0 (11.2 – 44.8)	
**BCLC stage**					
0 – A	91	55	31	41.0 (31.5 – 50.5)	**0.002**
B	46	18	10	6.0 (0 – 17.3)	
C	83	47	0	28.0 (11.2 – 44.8)	
**MELD score**					
< 11	92	61	34	41.0 (27.0 – 55.0)	0.060
≥ 11	78	41	18	25.0 (21.5 – 28.5)	
**Child-Pugh increase post-TACE**					
Absent	92	60	35	41.0 (33.6 – 48.3)	**0.003**
≥ 1 point	83	35	14	22.0 (10.3 – 33.6)	
**MELD increase post-TACE**					
Absent	94	39	44	44.0 (24.5 – 63.4)	**0.003**
≥ 1 point	80	43	17	25.0 (17.9 – 32.0)	
**Tumor response**					
CR	92	54	30	37.0 (25.4 – 48.6)	**0.048**
Non–CR	72	45	17	28.0 (17.8 – 38.2)	
**Recurrence**					
Absent	88	57	57	36.0 (12.1 – 59.9)	0.817
Present	94	72	55	40.0 (26.9 – 53.1)	
**Recurrence type**					
Local	95	63	32	42.0 (31.4 – 52.6)	0.312
Distant intrahepatic	100	53	39	44.0 (9.5 – 78.5)	
Local + distant intrahepatic	82	45	11	31.0 (12.1 – 49.9)	

Patients submitted to liver transplant (LT) were excluded from the analysis (34 patients). The assessment of tumor response was considered at 1 month after TACE. In the analysis of survival according to tumor response also patients not evaluable were excluded from the analysis.

*Abbreviations:* *HCV* hepatitis C virus, *TACE* transarterial chemoembolization, *AFP* Alpha-fetoprotein, *INR* international normalized ratio, *CPT* Child-Pugh-Turcotte score, *BCLC* Barcelona Clinic Liver Cancer, *MELD* Model for end stage liver disease, *CR* complete response, *PR* partial response, *PD* progressive disease, *PS* = Performance Statsus, *BCLC* Barcelona Clinic Liver Cancer.

P<0.05 are reported as bold numbers.

**Table 6:** **Liver function parameters of patients with tumor burden within Milan criteria (n = 135) on the basis of performance status**

Variable	PS-0 (n = 120)	PS-1 (n = 15)	*P*
**Ascites, n (%)**			1.000
Absent	98 (82)	12 (80)	
Present	22 (18)	3 (20)	
**Encephalopathy, n (%)**			0.300
Absent	118 (98)	14 (93)	
Slight	2 (2)	1 (7)	
**Serum total bilirubin, median (mg/dL) (range)**	1.39 (0.30 – 10.67)	1.23 (0.53 – 7.72)	0.817
**Serum albumin, median (g/dL) (range)**	3.60 (2.40 – 5.00)	3.60 (2.80 – 4.90)	0.596
**Serum INR, median (range)**	1.28 (1.00 – 1.97)	1.23 (1 – 1.86)	0.535
**Serum creatinine, median (range)**	0.92 (0.50 – 1.73)	0.78 (0.65 – 1.66)	0.115
**Hepatic function, n (%)**			0.784
CPT-A	74 (62)	10 (67)	
CPT-B	46 (38)	5 (33)	
**Hepatic function, n (%)**			0.655
CPT-A5	54 (45)	6 (40)	
CPT-A6	20 (17)	4 (27)	
CPT-B7	22 (18)	1 (7)	
CPT-B8	19 (16)	2 (13)	
CPT-B9	5 (4)	2 n	

*Abbreviations*: *CPT* Child-Pugh-Turcotte score, *INR* international normalized ratio.

All the variables in the univariate analysis with *P* <0.1 (Table 5) were entered into a Cox regression analysis, except for the CPT score and BCLC to avoid redundancy since the variables upon which they are built were already included in the analysis. After a conditional backward selection, tumor diameter beyond the Milan criteria (*P* = 0.015, OR = 3.0), lack of a complete radiological tumor response (*P* = 0.006, OR = 2.3), the presence of ascites before TACE (*P* = 0.021, OR = 2.3), AFP ≥14.5 ng/mL (*P* = 0.007, OR = 2.1) and a MELD score increase ≥1 point the day after TACE (*P* = 0.037, OR = 2.0) remained significant independent predictors of a worse survival.

## Discussion

Curative treatment is recommended as the first-line treatment for patients with single HCC regardless of tumor diameter [1, 7]. In clinical practice, however, patients with single tumors unfit for curative treatment are usually treated by TACE, based on clinical judgment. According to the current guidelines, TACE is the first line non-curative treatment for intermediate stage patients [1]. No evidence of a beneficial impact of TACE in patients with single HCC is reported in the guidelines since the trials upon which the guidelines are built [15], for the most part, included patients with multiple nodules of HCC. Accordingly, TACE is frequently performed outside the current treatment guidelines in a considerable percentage of patients with a single nodule, according to a “stage migration strategy” [16].

Only a few studies have evaluated the efficacy of TACE in patients with a single nodule [5, 17, 18] and a valid comparison with previous data reported in the literature is very difficult, due to the different criteria used for the evaluation of tumor response, TACE procedure, the selectivity of technique and the expertise of the radiological center. This fact led to the investigation of the overall survival and clinical determinants of survival in patients with a single nodule who represent approximately half (45%) of the total cohort of patients who underwent a first TACE cycle in our Interventional Radiology Unit (156/344) (Figure 1). This number is fully comparable to a very large Japanese series in which patients with single tumors were 46% of those who underwent TACE [5], and some other studies [14, 19] which showed high heterogeneity of patients routinely undergoing TACE, including 35-50% of patients with single tumors, even those <5 cm. Furthermore, the vast majority of the studies investigating the efficacy of TACE excluded patients with advanced liver disease, PVT and impaired PS; therefore, there was also no evidence of the impact of TACE in those categories of patients [20]. The allocation policy and the impact of TACE in patients with impaired liver function (namely CPT-B patients) has already been described [21] and, in the present study, the aim was to evaluate the impact of bland PVT and slight impairment of PS on overall survival after TACE.

The median overall survival of the entire patient population, after the exclusion of patients who underwent LT who were generally long term survivors, was 36.0 months with 1-, 3- and 5-years survival rates of 85%, 50% and 26%, respectively. These data are slightly lower than those observed in a large Japanese series [5] reporting 1-, 3- and 5-years survival rates of 91%, 66% and 53%, respectively in patients treated with TACE for a single nodule of HCC (even though no information regarding possible subsequent LT was reported). As expected, when comparing these results with those reported in the metanalysis of Llovet et al. (median survival of 20 months in patients who underwent TACE) in which the vast majority of patients had multinodular HCC [15], the median overall survival was considerably higher despite the large presence of CPT-B patients in our series. On the basis of survival analysis, TACE treatment indeed represents a valid therapeutic option for patients with single HCC who are not eligible for curative treatment, as has also been shown by recent series of BCLC-A patients from Barcelona and from Pisa [17, 22]. Such data also supported the use of the stage migration policy from the early to the intermediate HCC stage.

When assessing the clinical predictors of survival, tumor diameter >3 cm, and particularly >5 cm (beyond the MC), lack of complete radiological tumor response, AFP ≥14.5 ng/mL, the presence of ascites before TACE and a MELD increase ≥1 point the day after TACE were found to be independently associated with shorter survival at multivariate analysis. These data are in agreement with the fact that life expectancy depends not only upon tumor treatment efficacy, but also on the underlying severity of liver disease and patients with worsening hepatic function after TACE; with a MELD score increase ≥1 point, they are at risk of liver failure.

The presence of bland PVT in patients with HCC represents a challenging therapeutic issue. In recent decades, some authors [23] have considered the presence of PVT to be a contraindication for TACE due to the risk of liver function deterioration and hepatic infarct [24] but patients with PVT may not present technical and safety contraindications to TACE if a selective/superselective procedure is performed [20, 25]. In fact, more recent studies have demonstrated that TACE could be a safe treatment option for HCC patients with PV occlusion especially when performed in a selective manner [26], and that TACE could have a survival benefit over conservative treatment [27, 28]. In our Hospital, patients with bland thrombosis are candidates for TACE if they have preserved liver function, limited tumor burden, contraindications to other treatment and a selective approach is feasible. Interestingly, despite the limited number of patients with bland PVT (n = 17) who underwent TACE, our results showed that the presence of bland PVT, either lobar or segmental, has no negative impact on overall survival when TACE is performed with a selective or superselective approach.

The BCLC staging system includes the ECOG PS [12] evaluation regarding the assessment of tumor stage. In patients with HCC, the classic determination of PS is not able to differentiate between cancer- or cirrhosis- related symptoms [16] and the subjective assessment of “how the patient feels” can be related to cirrhosis as well as to cancer. In our study population, 15 patients with PS-1 were formally classified to be in the advanced stage (BCLC-C) (Table 2) but, since the tumor diameter was ≤5 cm, the likelihood of having cancer-related symptoms could be considered very low. Accordingly, these patients in BCLC-C had a significant and theoretically paradoxical better survival (28 months) than patients in the BCLC-B stage (6 months), as all the latter had large tumors (diameter >5 cm) (Tables 2, 5). It could be speculated that, in case of symptoms of uncertain tumor relation, the tumor burden should be considered the driving force for treatment allocation. On the other hand, considering patients with the same tumor burden (within the MC), PS-1, and consequently the same liver function, this certainly impacts survival so that BCLC-C patients (PS-1) had significantly worse survival with respect to BCLC 0-A patients (PS-0) (Figure 3).

To the best of our knowledge, the only data on tumor radiological response of conventional TACE in patients with single unresectable HCC is that of Malagari et al. [18]. Our study showed notably higher objective response rates (CR + PR) of 90% vs. 59.6% and superior rates of CR (64%) and PR (26%) (Table 3) as compared to the 4.8% CR and 54.8% PR reported by Malagari. Our data appeared consistent with those reporting per-nodule TACE efficacy (mimicking patients with a single nodule) [29] where similar CR and PR rates were reported (64% and 36%, respectively) and tumor diameter ≤5 cm was again found to be a statistical predictor of complete response [29].

We acknowledge that the response rate in our study might be overestimated since the assessment of radiological response was usually made with CT, which may underestimate the residual tumor due to the interference of Lipiodol [13]. The availability of MRI was not sufficient to offer this technique as a standard procedure after TACE to all patients. Nevertheless, our data are of current interest since, despite the introduction of TACE using drug-eluting beads [30, 31], clinical trials comparing TACE with drug-eluting beads and conventional TACE did not show significant differences in tumor response and overall survival [19, 32] and, nowadays, conventional TACE is still for the most part used.

A number of studies have demonstrated that the repetition of TACE increases tumor response and prolongs survival [16], but it is necessary to select the best TACE candidates who could benefit from treatment and eventually subsequent cancer retreatment in order to avoid overtreatment and detrimental effects on liver function. The issue of proper patient selection for retreatment after TACE has become more stringent in recent years due to the availability of alternative treatments such as sorafenib [33], and radioembolization [24]. To this end, the worsening of laboratory tests 24 hours after treatment was evaluated and a significant modification in serum albumin, bilirubin, the INR and the MELD score after TACE treatment was documented (Figure 2). As expected, it was also found that patients with a CPT score increase ≥1 point were more likely to undergo a single TACE cycle vs. multiple cycles (70% vs. 30%, *P* = 0.006) Furthermore, both a CPT and a MELD score ≥1 point increase were found to be associated with a significantly worse prognosis (Table 4). Such findings do not affect the initial choice of recommending TACE, but seem to alert clinicians to consider the risk that patients will be no more candidate for future repeated TACE in case of early CPT score worsening after the procedure, deserving an even more careful assessment of treatment strategy.

## Conclusion

In conclusion, our results showed TACE to be an effective curative treatment in patients with a single nodule of HCC who were not eligible for curative treatment, supporting the strategy of stage migration for early single HCC. Bland PVT has no major impact on survival and a slight impairment of PS (PS-1) most likely attributable to cirrhosis in patients with a tumor burden within the Milan criteria should not preclude the use of TACE. This confirmed the fundamental role of individual clinical judgment in the treatment of HCC.
